# Age and Colorectal Cancer Outcomes: A Comparative Analysis Between Patients Younger and Older than 70 Years

**DOI:** 10.3390/curroncol33020100

**Published:** 2026-02-04

**Authors:** Oswaldo Moraes Filho, Bruno Augusto Alves Martins, André Araújo de Medeiros Silva, Romulo Medeiros de Almeida, Antonio Carlos Nobrega dos Santos, Camila Oliveira Barbosa, Flávia Berford Leão dos Santos Gonçalves de Oliveira, Tuane Colles, Wilmar Junio Pereira Araújo, João Batista de Sousa

**Affiliations:** 1 Coloproctology Service, University Hospital of Brasília, Setor de Grandes Áreas Norte 605, Asa Norte, Brasília 70840-901, DF, Brazil; bruno.augusto@ebserh.gov.br (B.A.A.M.); amdreams2@gmail.com (A.A.d.M.S.); romulo.almeida@ebserh.gov.br (R.M.d.A.); acnobrega@unb.br (A.C.N.d.S.); barbosacamila@yahoo.com.br (C.O.B.); fberford@terra.com.br (F.B.L.d.S.G.d.O.); tuane.colles@ebserh.gov.br (T.C.); wilmar.araujo@ebserh.gov.br (W.J.P.A.); sousajb@unb.br (J.B.d.S.); 2 School of Medicine, University of Brasília, Brasília 70910-900, DF, Brazil

**Keywords:** colorectal neoplasms, aged, survival analysis, treatment outcome, age factors

## Abstract

Colorectal cancer is predominantly a disease of older adults, yet age-related treatment decisions remain controversial. While chronological age is often used as a criterion for surgical eligibility, it remains unclear whether age alone is an independent predictor of surgical and oncological outcomes. This study evaluated whether age is a significant determinant of outcomes in colorectal cancer patients undergoing surgical resection. This retrospective comparative study analyzed 262 patients (193 younger than 70 years, 69 aged ≥ 70 years) diagnosed with colorectal cancer stages I–IV between 2014 and 2021 at a tertiary single center. Survival analysis was conducted using Kaplan–Meier method and Cox proportional hazards regression. Elderly patients had higher ASA classification (*p* = 0.0270), higher hypertension prevalence (*p* < 0.0001), higher ICU admission rates (50.7% vs. 21.2%, *p* < 0.0001), and longer hospital stays (12.6 vs. 7.5 days, *p* = 0.0016). However, elderly patients presented with earlier-stage disease (Stage I + II: 64.2% vs. 46.1%, *p* = 0.0108). After adjustment for confounding factors, age did not significantly impact overall survival (HR = 1.33; 95%CI: 0.54–3.26; *p* = 0.5375) or disease-free survival (HR = 1.61; 95%CI: 0.79–3.29; *p* = 0.1939). Despite differences in clinical presentation and pathological findings, age itself was not an independent predictor of survival outcomes. These findings suggest that treatment decisions in elderly colorectal cancer patients should be informed by individual patient physiology and disease stage rather than chronological age alone.

## Simple Summary

Colorectal cancer is a major health concern worldwide, and its treatment becomes more complex as patients age. Many doctors hesitate to operate on elderly patients due to concerns about worse outcomes related to age and multiple health conditions. We studied 262 colorectal cancer patients in Brazil, comparing those under 70 years old with those 70 and older. We found that elderly patients presented with earlier-stage cancers, despite having more health problems. After surgery, their long-term survival was comparable to that of younger patients when we accounted for all relevant factors. These findings suggest that chronological age alone should not prevent elderly patients from receiving surgery. Instead, doctors should evaluate each older patient individually to determine if surgery is appropriate, rather than making decisions based solely on age. These results support a more personalized approach to treating elderly colorectal cancer patients.

## 1. Introduction

Colorectal cancer (CRC) is among the most diagnosed cancers worldwide, the third most incident, and the second most deadly cancer, according to GLOBOCAN data The estimated new cases and deaths of CRC were approximately 2.0 million and 1.0 million in the year 2022 across the world [1]. In Brazil, it is the second most frequently diagnosed tumor in both men and women and represents a significant public health issue [2].

Colorectal cancer is mainly a disease of older patients; approximately 60% of cases occur in individuals aged 65 years or older [3]. Brazil faces a unique epidemiological challenge with colorectal cancer. Although CRC incidence is rising fastest among younger adults, the rapid aging of the Brazilian population means that the absolute number of older adults with colorectal cancer is projected to increase substantially in coming decades, while younger adults will account for a growing share of new cases [2,4]. Unlike many developed nations where CRC incidence has plateaued or declined, Brazil continues to experience rising incidence rates, particularly in the South and Southeast regions, with regional variations reflecting differences in healthcare access, screening practices, and socioeconomic factors [4,5]. Additionally, Brazil is experiencing rapid population aging, with the proportion of adults aged 60 years and older projected to increase from approximately 12% in 2010 to over 25% by 2050 [6]. This dual epidemiological burden—both an increase in early-onset disease and an increase in elderly-onset disease—creates a complex clinical landscape that demands evidence-based approaches to treatment decision-making across all age groups. This aging population creates distinctive challenges for healthcare systems and for clinicians, who need to treat elderly patients with unique physiological characteristics, multiple comorbidities, and possibly differing tumor biology in comparison to younger patients [7]. In the Brazilian context, where healthcare resources are often limited, understanding how age influences surgical outcomes and treatment decisions is particularly important for optimizing the allocation of resources and ensuring equitable access to curative treatment.

The surgical resection is still the mainstay of curative treatment of CRC. But the optimal treatment for elderly colorectal cancer patients remains controversial. Older age has previously been correlated with elevated postoperative morbidity and mortality, which may ultimately result in older patients being undertreated [8]. While elderly patients with colorectal cancer have been reported to present with more advanced disease and higher rates of emergency presentation, it remains unclear whether age itself is an independent predictor of outcomes when comparing patients with similar disease stage who undergo planned surgical resection. This distinction motivated our study, which focuses exclusively on patients undergoing elective surgery to isolate the effect of age independent of presentation type [9,10].

The effect of age on oncological results after colorectal cancer surgery is still a matter of debate. While some have reported poorer survival in elderly patients [11,12], others have found similar cancer-specific survival after correcting for comorbidities and stage [13,14]. These contradictory findings have demonstrated that it is not an easy task to clarify how age truly affects outcomes of colorectal cancer, and that it is necessary to perform a comprehensive analysis that incorporates several confounding variables.

Moreover, improvements in surgical techniques, surgical perioperative care, and anesthetics have led to improved outcomes for all patients who underwent a colorectal cancer operation, even the elderly ones [15]. Morbidity and mortality were still reduced in recent times due to minimally invasive procedures, an intensified recovery schedule, and better patient selection [16,17]. Nevertheless, whether these advancements have been translated into an advantage for elderly patients, as compared with younger patients, is not clear.

Furthermore, the association between age and pathological features of colorectal tumors is appealing. Several studies have indicated that elderly patients could exhibit distinct tumor features such as location, the type of histology, and molecular features [18,19]. These differences could potentially influence treatment response and oncological outcomes, but the evidence remains inconclusive.

Given these considerations, there is a need for comprehensive studies that evaluate the impact of age on multiple aspects of colorectal cancer, including epidemiological characteristics, clinical presentation, surgical outcomes, pathological features, and oncological results. Such analyses would provide valuable insights for optimizing the management of elderly patients with colorectal cancer and developing age-appropriate treatment strategies.

The objective of the present study was to evaluate the epidemiological data, clinical presentation, surgical treatment, pathological analysis, and oncological outcomes of colorectal cancer patients under 70 years of age when compared with those over 70 years in a tertiary university hospital from Brazil, where a prospectively maintained database was used. By addressing these various facets, we hope to offer a well-rounded view of the influence of age on colorectal cancer and to contribute to evidence-based strategies for treating this ever-expanding group of patients.

## 2. Materials and Methods

### 2.1. Study Design and Population

This study is a retrospective cohort analysis conducted at a single center, utilizing prospective data from patients with colorectal cancer who underwent surgery between January 2014 and December 2021 at the University Hospital of Brasília, Brazil. The research adhered to local ethical guidelines and was approved by the institutional review board of the University of Brasília (approval number 63179920.6.0000.5558, dated 30 January 2023).

The study included patients aged 18 years and older diagnosed with histologically confirmed adenocarcinoma of the colon or rectum who underwent elective surgical resection with curative intent. Exclusion criteria encompassed emergency surgery and patients with hereditary colorectal cancer syndromes (familial adenomatous polyposis and Lynch syndrome), synchronous tumors, inflammatory bowel disease, and previous colonic resection.

### 2.2. Data Collection

Data were collected from a prospectively maintained database at the coloproctology service of the University Hospital of Brasília. For each patient, the following information was recorded:

Demographic data: Age, sex, body mass index (BMI), American Society of Anesthesiologists (ASA) classification that presents as I + I and III + IV, smoking status, and comorbidities (diabetes mellitus and hypertension).

Clinical presentation: The patient presents with abdominal pain, changes in bowel habits, weight loss, bleeding, and a time interval between symptom onset and diagnosis (TISD). The TISD is the duration from the initial appearance of any colorectal cancer-related sign or symptom to the establishment of a diagnosis.

Surgical data: Surgical approach (laparoscopic or open), conversion to open surgery, length of hospital stay, operative mortality, reoperation rates, and intensive care unit (ICU) admission. Operative mortality was defined as death from any cause within 30 days of surgery or within 30 days of the first procedure for patients who underwent reoperation.

Pathological data: Tumor location, TNM staging according to the 8th edition of the American Joint Committee on Cancer (AJCC), T presents as T1 + T2 and T3 + T4, and N presents as N0 and N1 + N2, oncological staging presents as I + II, III e IV, histological type (well-differentiated + moderately differentiated and poorly differentiated), lymph node yield, perineural invasion, and angiolymphatic invasion.

Oncological outcomes: Disease-free survival (DFS) and overall survival (OS).

### 2.3. Study Groups and Variables

Patients were divided into two groups based on age: younger than 70 years (<70) and 70 years or older (≥70). The cutoff age of 70 years was chosen based on previous literature and clinical relevance in geriatric oncology [20,21].

The primary outcomes were overall survival (OS) and disease-free survival (DFS). OS was defined as the time from surgery to death from any cause, while DFS was defined as the time from surgery to disease recurrence or death, whichever occurred first. Secondary outcomes included clinical presentation, surgical, and pathological outcomes.

### 2.4. Statistical Analysis

Categorical variables were presented as frequencies and percentages, and continuous variables as means and standard deviations. For comparison between age groups, the chi-square test was used for categorical variables, and the t-student or Mann–Whitney test for continuous variables, depending on the normality of distribution.

Poisson regression models with robust variance (log-linear) were employed to analyze the association between age and clinical presentation, surgical outcomes, and pathological staging, controlling for the effects of covariates such as sex, age, BMI, smoking status, diabetes mellitus, hypertension, and ASA classification. Poisson regression was chosen because it provides a better estimate of prevalence ratios, which represent more meaningful effect measures for cross-sectional studies [22].

Cumulative logistic regression models [23] were used to analyze the association between age and final stage (I and II; III; IV), controlling for the effects of covariates such as sex, tumor location, BMI, and smoking status. In this model, the stage categories were considered in ascending order.

Cox regression models were used to analyze the association between age and overall and disease-free survival, adjusted for a set of clinical covariates. Initially, simple Cox regression models were fitted for each covariate, and those with a p-value less than 0.25 [24] were included in the multiple Cox regression analysis. Subsequently, adjustments to these variables were made through a process of removing/including variables. Only covariates with *p* < 0.05 remained in the final model. Finally, the independent variable of interest, age, was introduced to verify its association with time until death or recurrence after adjusting for possible confounders. Hazard ratios (HR) and their respective 95% confidence intervals were calculated.

Overall survival and disease-free survival curves for patients younger than 70 years and those 70 years or older were constructed and compared using the log-rank test.

All analyses were performed using SAS 9.4 (SAS Institute Inc., Cary, NC, USA). The significance level was set at 0.05.

## 3. Results

### 3.1. Demographic Characteristics

A total of 262 patients with a confirmed histologic diagnosis of colorectal adenocarcinoma were evaluated. Among them, 193 (73.6%) were younger than 70 years, and 62 (26.3%) were 70 years or older.

Table 1 shows the distribution of patients according to demographic variables by age group. No significant differences were observed between age groups regarding sex distribution (*p* = 0.5995), smoking status (*p* = 0.5907), or BMI (*p* = 0.9669). However, patients aged 70 years or older had a significantly higher prevalence of ASA classification III and IV compared to younger patients (37.7% vs. 23.8%, *p* = 0.0270). Similarly, the prevalence of hypertension was significantly higher in elderly patients (74.6% vs. 45.6%, *p* < 0.0001). There was a trend toward higher prevalence of diabetes mellitus in elderly patients, although this did not reach statistical significance (29.9% vs. 18.7%, *p* = 0.0547).

### 3.2. Clinical Presentation

Table 2 presents the distribution of patients according to clinical presentation variables by age group. No statistically significant differences were found between age groups regarding the presence of weight loss (*p* = 0.1886), changes in bowel habits (*p* = 0.6281), bleeding (*p* = 0.2831), abdominal pain (*p* = 0.3737), or time interval between symptom onset and diagnosis (*p* = 0.3602).

In the Poisson regression analysis, both unadjusted and adjusted for sex, tumor location, BMI, smoking status, and diabetes mellitus, no significant associations were found between age and any clinical presentation variables. The adjusted prevalence ratios were 1.13 (95% CI: 0.86–1.51, *p* = 0.3656) for changes in bowel habits, 0.87 (95% CI: 0.64–1.17, *p* = 0.3546) for abdominal pain, 0.81 (95% CI: 0.59–1.11, *p* = 0.1939) for weight loss, 0.92 (95% CI: 0.73–1.17, *p* = 0.5213) for bleeding, and 1.16 (95% CI: 0.91–1.48, *p* = 0.2229) for time interval between symptom onset and diagnosis ≥6 months.

### 3.3. Surgical Outcomes

Table 3 shows the distribution of patients according to perioperative variables by age group. The surgical approach (laparoscopic vs. open) was similar between age groups (*p* = 0.7365), with laparoscopic surgery performed in 60.9% of elderly patients and 58.6% of younger patients. No significant differences were observed in conversion rates (10.1% vs. 7.8%, *p* = 0.5419), reoperation rates (15.9% vs. 14.0%, *p* = 0.6926), or operative mortality (14.5% vs. 9.3%, *p* = 0.2332).

However, significant differences were found in ICU admission rates and length of hospital stay. The percentage of patients admitted to the ICU was significantly higher among those aged 70 years or older compared to younger patients (50.7% vs. 21.2%, *p* < 0.0001). Similarly, the mean length of hospital stay was significantly longer in elderly patients (12.6 ± 15.5 days vs. 7.5 ± 5.7 days, *p* = 0.0016).

In the Poisson regression analysis adjusted for sex, tumor location, BMI, smoking status, diabetes mellitus, hypertension, and ASA classification, the prevalence of ICU admission remained significantly higher in elderly patients (PR = 1.89, 95% CI: 1.32–2.71, *p* = 0.0005). Likewise, the prevalence of hospital stay ≥4 days was significantly higher in elderly patients (PR = 1.11, 95% CI: 1.02–1.21, *p* = 0.0112). No significant associations were found between age and conversion to open surgery, operative mortality, or reoperation rates.

### 3.4. Pathological Findings

Table 4 presents the distribution of patients according to pathological variables by age group. No significant differences were observed between age groups regarding tumor location (colon vs. rectum, *p* = 0.7180), T stage (T1 + T2 vs. T3 + T4, *p* = 0.7870), angiolymphatic invasion (*p* = 0.7806), perineural invasion (*p* = 0.3618), histological type (*p* = 0.1926), or adjuvant chemotherapy status (*p* = 0.4343).

However, significant differences were found in the N stage and overall tumor stage. The percentage of patients with N0 stage was significantly higher among those aged 70 years or older compared to younger patients (67.2% vs. 47.6%, *p* = 0.0067). Similarly, the percentage of patients with stage I + II disease was significantly higher in elderly patients (64.2% vs. 46.1%, *p* = 0.0108).

In the cumulative logistic regression analysis adjusted for sex, tumor location, BMI, and smoking status, the odds of an elderly patient being in a lower category of final stage (I and II vs. III vs. IV) were 95% higher than that of a younger patient (OR = 1.95, 95% CI: 1.11–3.45, *p* = 0.0207). Similarly, in the adjusted Poisson regression analysis, elderly patients had a 41% higher prevalence of N0 stage compared to younger patients (PR = 1.41, 95% CI: 1.12–1.77, *p* = 0.0030).

### 3.5. Survival Analysis

**Figure 1:**
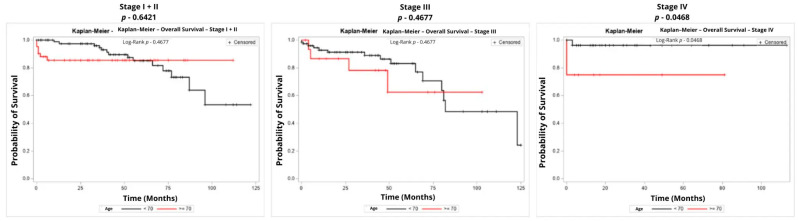
Kaplan–Meier Curves for Overall Survival by Stage.

Kaplan–Meier survival analysis was performed to compare overall survival and disease-free survival between age groups, stratified by tumor stage. For patients with stage I + II disease, no significant difference was observed in overall survival between age groups (*p* = 0.6421). Similarly, for patients with stage III disease, overall survival did not differ significantly between age groups (*p* = 0.4677). However, for patients with stage IV disease, overall survival was significantly better in patients younger than 70 years compared to elderly patients (*p* = 0.0468), although this finding should be interpreted with caution due to the small number of cases evaluated (*n* = 37). Table 5 shows crude and adjusted hazard ratios according to Cox regression models for overall survival, and Figure 1 shows the Kaplan-Meier Curves for overall survival of stages I + II, III, and IV, respectively.

Regarding disease-free survival, no significant differences were observed between age groups for patients with stage I + II disease (*p* = 0.5804) or stage III disease (*p* = 0.8758).

In the Cox regression analysis adjusted for surgical approach and BMI, age was not significantly associated with overall survival (HR = 1.33, 95% CI: 0.54–3.26, *p* = 0.5375). Similarly, in the Cox regression analysis adjusted for BMI and CEA level, age was not significantly associated with disease-free survival (HR = 1.61, 95% CI: 0.79–3.29, *p* = 0.1939).

**Figure 2:**
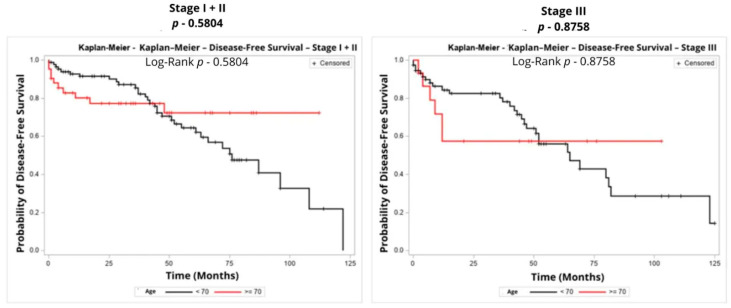
Kaplan–Meier Curves for Disease-Free Survival by Stage.

Table 6 shows crude and adjusted hazard ratios according to Cox regression models for disease-free survival, and Figure 2 shows the Kaplan–Meier Curves for disease-free survival of stages I + II and III, respectively.

## 4. Discussion

This study compared the epidemiological data, clinical presentation, surgical results, pathology, and oncological outcome of patients under 70 years of age with colorectal cancer and those 70 years or older submitted to surgery in a tertiary university service were compared in the present study. Our findings provide valuable insights into the impact of age on various aspects of colorectal cancer management and outcomes.

The results showed that the older patients, more than the young, have shown a significant association with ASA Grade III and IV, and hypertension. These observations are in line with previous reports on increased comorbidity load in elderly CRC patients [25,26]. Jafari et al. retrospectively reviewed the American College of Surgeons National Surgical Quality Improvement Program (ACS-NSQIP). They observed that cardiovascular comorbidities, such as hypertension, were significantly more prevalent among patients who were 70 years of age or older when compared with younger patients [27]. Similarly, Aquina et al. found that patients aged ≥65 years had more comorbid conditions that might affect surgical outcomes and survival compared to younger patients [11]. The predominance of comorbidities in older patients holds great significance for perioperative management and decision-making. Preoperative risk stratification and risk management by comorbidity optimization are significant for better postoperative outcomes in this patient population [28]. Furthermore, multiprofessional interventions, such as those including geriatricians, anesthesiologists, and oncologists, might be advantageous for elderly patients with colorectal cancer [29].

Interestingly, our study showed no clinical differences between age groups, an observation that contrasts with some previous findings that elderly patients have different presentations or even more severe diseases [30,31]. For instance, Patel et al. found that older patients were more commonly presented with emergency symptoms, including obstruction and perforation, than were younger patients, and that younger patients were highly associated with hematochezia and change in bowel habit [32]. The lack of significant differences in clinical presentation in our study may be attributed to several factors. First, we collected data on patients who underwent surgery; our cohort may not be a true reflection of the general patient population, especially for the subset that had more severe comorbidities or advanced disease that had been ruled out for a surgical option. Second, enhanced awareness of colorectal cancer symptoms and improved access to health care may have resulted in earlier detection throughout all age groups. Third, regional variations in healthcare systems and patient behaviors could influence the pattern of clinical presentation.

Regarding surgical outcomes, significant differences between age groups were observed in our study. The type of operation, the rate of conversion, the reoperation rate, and the rate of surgical death were all comparable in the elderly and in the young patients; however, the elderly patients were more frequently admitted to the ICU and had longer hospitalization. These findings are consistent with previous papers, which have found that elderly patients with colorectal cancer surgery may have more prolonged recovery and greater resource use [27,33]. The reason for higher ICU admission rates in aged patients is multifactorial. Despite higher baseline comorbidity burden (ASA III–IV: 37.7% vs. 23.8%; hypertension: 74.6% vs. 45.6%), even after multivariable adjustment for these measured comorbidities, elderly patients remained significantly more likely to be admitted to the ICU (PR = 1.89, *p* = 0.0005). This suggests that the higher ICU admission in elderly patients is not fully explained by measured comorbidities alone, but may reflect age-related physiological differences, clinical decision-making influenced by age itself, or unmeasured aspects of frailty. A more conservative attitude for post-operation management may be held for elderly patients, but the specific clinical criteria and reasoning for ICU admission decisions were not available for detailed analysis in this retrospective study. Prolonged hospital stays could be attributed to slower recovery, higher incidence of minor complications not requiring reoperation, or social factors affecting discharge planning [34]. However, we are encouraged by the similarity of the operative mortality and reoperation rates between the age groups, and with good patient selection and perioperative care, it indicates that the elderly patient can undergo surgery for colorectal cancer safely.

Modern advances in surgery, anesthesia, and perioperative care have led to improved outcomes in elderly individuals. In those patients, minimally invasive procedures, such as laparoscopy and robotics, have demonstrated less morbidity and more rapid recovery [35,36]. Our institution has progressively adopted key perioperative care principles aligned with ERAS (Enhanced Recovery After Surgery) guidelines, including enhanced anesthesia techniques, minimally invasive approaches, and optimized postoperative management, although we do not have a formally certified ERAS protocol in place. These practices have shown potential benefits in terms of length of stay and postoperative complications [37]. Our observation that the majority of elderly patients received laparoscopic surgery (60.9%) could be explained by the introduction of these new techniques by this institution.

The most interesting finding in our research was that the elderly group had a higher proportion of N0 stage and early stage of the tumor (I + II) than the youth group. This finding contrasts with earlier reports where it was found that old patients have more advanced disease [38,39]. Several factors could explain this finding. First, there might be biological differences in tumor behavior between age groups. Some studies have suggested that colorectal tumors in elderly patients may have distinct molecular features and potentially less aggressive behavior) [40,41,42]. Second, selection bias may exist because elderly patients with more advanced disease were less likely to receive surgical resection due to a perceived increased risk or minimal benefits. Third, variation in screening and healthcare-seeking behavior between age groups might affect stage at diagnosis.

The higher proportion of early-stage diseases in the older population has a significant influence on treatment options. It implies that the chronological age should not be an obstacle for curative-intent operative intervention in older colon cancer patients, given that most patients present with potentially curable disease. Furthermore, it highlights the importance of appropriate staging and comprehensive geriatric assessment to guide treatment decisions in this population [43].

Our analysis combined colon and rectal cancers in evaluating age-related surgical outcomes. While these anatomical sites have distinct treatment strategies in certain contexts, they share a common embryological origin and are frequently analyzed together in large epidemiological studies examining age-related outcomes in colorectal cancer. This approach is consistent with major epidemiological studies, such as Gabriel et al. (2018), which combined 488,121 patients with colon cancer and 181,909 with rectal cancer in examining age-related differences in disease characteristics and survival outcomes [44]. Our combined analysis was also justified by sample size considerations, as stratifying by both age and tumor location would result in subgroups too small for robust statistical analysis, particularly for elderly patients with rectal cancer (*n* = 19).

An important consideration in interpreting our findings is the inherent selection bias in surgical cohorts, particularly in geriatric populations. Selection bias, also referred to as “healthy survivor bias”, refers to the phenomenon that only certain individuals survive to reach old age and are deemed suitable for major surgery. In our study, elderly patients with more advanced diseases or significant functional impairment were less likely to be offered surgical resection, creating a natural selection of elderly candidates for surgery. This is reflected in our findings that elderly patients presented with earlier-stage disease (64.2% vs. 46.1% with stages I + II, *p* = 0.0108), while simultaneously carrying higher comorbidity burden (ASA III–IV: 37.7% vs. 23.8%, *p* = 0.0270). This apparent paradox—higher comorbidities but earlier disease—is characteristic of selection bias in surgical cohorts. It contextualizes them appropriately: our results demonstrate that when elderly patients are carefully selected for surgical resection, they achieve oncological outcomes comparable to those of younger patients, even in the presence of a substantial comorbidity burden. This distinction is clinically important, as it suggests that chronological age alone should not serve as a barrier to curative-intent surgery in appropriately selected elderly patients with colorectal cancer. This pattern of healthier elderly surgical candidates is well-documented in the colorectal cancer literature, confirming that selection bias is a characteristic feature of surgical outcome studies in this population [45].

Our survival analysis also demonstrated that age was not an independent predictor for the overall survival and disease-free survival in the multivariate analysis. This finding is particularly relevant as it demonstrates that advanced age alone does not inherently confer worse oncological outcomes in colorectal cancer. Similar results have been reported in several prior studies. For instance, Mima et al. did not identify age as an independent predictor of cancer-specific survival among stage I-III colorectal cancer patients after correcting for comorbidity and other prognostic variables [13]. Similarly, Aquina et al. found that cancer-specific mortality was comparable between elderly and younger patients after adjusting for stage and treatment parameters. However, general mortality was higher in the aged patients [10].

Our analysis revealed a statistically significant difference in overall survival for patients with stage IV disease (*p* = 0.0468), with younger patients demonstrating superior survival outcomes compared to elderly patients. Although this finding warrants cautious interpretation given the limited number of stage IV cases (*n* = 37), it represents an important observation that merits discussion.

The poorer survival observed in elderly patients with metastatic colorectal cancer may be attributable to several factors. One possible explanation is the use of less aggressive systemic therapy in elderly patients with metastatic disease or higher non-cancer-related mortality in such patients [46]. Recent studies have demonstrated that elderly patients with metastatic CRC receive fewer cycles of chemotherapy and are less likely to receive combination regimens (doublet or triplet therapy) compared to younger patients [47,48]. However, recent evidence suggests that carefully selected elderly patients with good functional status can tolerate and benefit from standard chemotherapy regimens comparable to those of younger patients [49].

It is important to note that the overall finding of comparable survival outcomes between age groups in our cohort suggests that age alone is not an independent predictor of outcomes when patients are appropriately selected for surgery. This observation underscores the clinical relevance of an individualized approach to treatment decision-making in elderly patients with colorectal cancer. Fit elderly patients may achieve similar oncological outcomes as younger patients, favoring an individualized attitude towards treatment decisions based on physical status, comorbidities, and patient preference rather than chronological age itself [46,50]. This highlights the critical importance of comprehensive geriatric assessment in treatment planning for elderly patients with advanced colorectal cancer [51].

### 4.1. Strengths and Limitations

This study has several strengths, including the comprehensive assessment of multiple aspects of colorectal cancer (epidemiological, clinical, surgical, pathological, and oncological), the use of robust statistical methods to adjust for confounding factors, and the inclusion of patients from a real-world clinical setting in a middle-income country.

However, some limitations should be acknowledged. First, the study’s retrospective nature introduces potential selection bias, as only patients who underwent surgery were included. Second, the single-center design may limit the generalizability of the findings to other settings with different patient populations or treatment approaches. Third, data on specific postoperative complications beyond ICU admission, reoperation, and mortality were not available for analysis. Fourth, our assessment was limited to hypertension, diabetes mellitus, and ASA classification, without formal geriatric-specific measures such as frailty evaluation, functional status, or cognitive function. These measures may be more relevant than age alone in predicting outcomes and should be incorporated in future prospective studies. Fifth, as a single-center study, our findings may not reflect regional variations in healthcare systems, patient populations, or treatment approaches that could influence outcomes in elderly colorectal cancer patients.

Finally, the relatively short follow-up period may have limited the assessment of long-term oncological outcomes.

### 4.2. Future Directions

Future research should focus on prospective studies incorporating comprehensive geriatric assessments to identify better elderly patients who would benefit most from surgical intervention. Additionally, investigating the impact of enhanced recovery protocols and minimally invasive approaches specifically in elderly patients with colorectal cancer would provide valuable insights for optimizing perioperative care in this population. Finally, exploring the molecular and biological characteristics of colorectal tumors across different age groups could help elucidate potential differences in tumor behavior and treatment response.

## 5. Conclusions

This study demonstrated that, although elderly patients (≥70 years) with colorectal cancer present with a higher comorbidity burden and require more intensive postoperative care, they paradoxically present with earlier-stage disease. Notably, after adjustment for confounding factors, chronological age did not emerge as an independent predictor of overall survival or disease-free survival. demonstrate that age-based treatment decisions should be informed by individual patient physiology and disease stage rather than chronological age alone and reinforce the necessity for an individualized approach to therapeutic decision-making based on physiological status, comorbidities, and patient preferences rather than chronological age alone. The higher prevalence of early-stage disease in elderly patients suggests that many may benefit from curative-intent surgical intervention.

## Figures and Tables

**Table 1:** Demographic and Clinical Characteristics by Age Group.

Characteristic	Total (*n* = 262)	Age < 70 Years (*n* = 193)	Age ≥ 70 Years (*n* = 69)	*p*-Value
**Sex, *n* (%)**				0.600
Female	141 (53.8)	102 (52.9)	39 (56.5)	
Male	121 (46.2)	91 (47.1)	30 (43.5)	
**ASA score, *n* (%)**				0.027
I–II	190 (72.5)	147 (76.2)	43 (62.3)	
III–IV	72 (27.5)	46 (23.8)	26 (37.7)	
**Smoking, *n* (%)**				0.591
No	228 (87.7)	168 (87.0)	60 (89.6)	
Yes	32 (12.3)	25 (13.0)	7 (10.4)	
**Diabetes mellitus, *n* (%)**				0.055
No	204 (78.5)	157 (81.3)	47 (70.1)	
Yes	56 (21.5)	36 (18.7)	20 (29.9)	
**Hypertension, *n* (%)**				<0.001
No	122 (46.9)	105 (54.4)	17 (25.4)	
Yes	138 (53.1)	88 (45.6)	50 (74.6)	
**Tumor location, *n* (%)**				0.718
Colon	184 (70.2)	134 (70.2)	50 (72.5)	
Rectum	76 (29.8)	57 (29.8)	19 (27.5)	
BMI, kg/m^2^, mean ± SD	25.27 ± 4.22	25.53 ± 4.16	25.68 ± 4.43	0.967

Data presented as *n* (%) for categorical variables and mean ± standard deviation for continuous variables. *p*-values calculated using the Chi-square test for categorical variables and the Mann–Whitney test for continuous variables. BMI: Body Mass Index; ASA: American Society of Anesthesiologists; SD: Standard Deviation.

**Table 2:** Distribution of patients according to clinical presentation variables by age group and adjusted prevalence ratios.

Variables	Total	Age		*p*-Value	Adjusted Prevalence Ratio (95% CI) *	*p*-Value
		<70	≥70			
**Changes in Bowel Habits**				0.6281		
No	123	93 (51.96)	30 (48.39)		1.13 (0.86, 1.51)	0.3656
Yes	118	86 (48.04)	32 (51.61)			
**Abdominal Pain**				0.3737		
No	119	86 (47.51)	33 (54.10)		0.87 (0.64, 1.17)	0.3546
Yes	123	95 (52.49)	28 (45.90)			
**Weight Loss**				0.1886		
No	119	85 (47.75)	34 (57.63)		0.81 (0.59, 1.11)	0.1939
Yes	118	93 (52.25)	25 (42.37)			
**Bleeding**				0.2831		
No	100	71 (39.01)	29 (46.77)		0.92 (0.73, 1.17)	0.5213
Yes	144	111 (60.99)	33 (53.23)			
**TISD (months)**	9.44 ± 11.47	9.35 ± 11.93	9.70 ± 10.07	0.3602	1.16 (0.91, 1.48) ^†^	0.2229

Values expressed as frequency (%) or mean ± standard deviation. * Adjusted for sex, tumor location, BMI, smoking status, diabetes mellitus, hypertension, and ASA classification ^†^ Adjusted prevalence ratio for TISD (≥6 vs. <6 months). *p*-value calculated using the Chi-square test or the Mann–Whitney test. Adjusted for sex, tumor location, BMI, smoking status, and diabetes mellitus. TISD—Time interval between Symptoms and Diagnosis.

**Table 3:** Distribution of patients according to surgical outcomes by age group and adjusted prevalence ratios.

Variables	Total	Age		*p*-Value	Adjusted Prevalence Ratio (95% CI) *	*p*-Value
		<70	≥70			
**Surgical Approach**				0.7365		
Open	107	80 (41.45)	27 (39.13)		1.07 (0.86, 1.32) ^†^	0.5537
Laparoscopic	155	113 (58.55)	42 (60.87)			
**Conversion**				0.5419		
No	240	178 (92.23)	62 (89.86)		1.07 (0.48, 2.37)	0.8706
Yes	22	15 (7.77)	7 (10.14)			
**Reoperation**				0.6926		
No	224	166 (86.01)	58 (84.06)		0.82 (0.42, 1.60)	0.5672
Yes	38	27 (13.99)	11 (15.94)			
**ICU Admission**				<0.0001		
No	186	152 (78.76)	34 (49.28)		1.89 (1.32, 2.71)	0.0005
Yes	76	41 (21.24)	35 (50.72)			
**Operative Mortality**				0.2332		
No	234	175 (90.67)	59 (85.51)		0.94 (0.41, 2.18)	0.8948
Yes	28	18 (9.33)	10 (14.49)			
**Length of Hospital Stay (days)**	8.85 ± 9.57	7.49 ± 5.65	12.64 ± 15.54	0.0016	1.11 (1.02, 1.21) ^‡^	0.0112

Values expressed as frequency (%) or mean ± standard deviation. * Adjusted for sex, tumor location, BMI, smoking status, diabetes mellitus, hypertension, and ASA classification. ^†^ Adjusted prevalence ratio for Surgical Approach (Laparoscopic vs. Open). ^‡^ Adjusted prevalence ratio for Length of Hospital Stay (≥4 vs. <4 days). Adjusted for sex, tumor location, BMI, smoking status, diabetes mellitus, hypertension, and ASA classification. *p*-value calculated using the Chi-square test or the Mann–Whitney test. ICU—Intensive Care Unit.

**Table 4:** Distribution of patients according to pathological variables by age group and adjusted prevalence ratios.

Variables	Total	Age		*p*-Value	Adjusted Prevalence Ratio (95% CI) *	*p*-Value
		<70	≥70			
**Clinical Staging**				0.6157		
M0	227	166 (86.01)	61 (88.41)		-	-
M1	35	27 (13.99)	8 (11.59)			
**T Stage**				0.7870		
T1 + T2	66	50 (26.32)	16 (24.62)		1.01 (0.86, 1.20) ^†^	0.8788
T3 + T4	189	140 (73.68)	49 (75.38)			
**N Stage**				0.0067		
N0	132	89 (47.59)	43 (67.19)		1.41 (1.12, 1.77)	0.0030
N1 + N2	119	98 (52.41)	21 (32.81)			
**Tumor Stage**				0.0108		
I + II	132	89 (46.11)	43 (64.18)		1.95 (1.11, 3.45) ^‡^	0.0207
III + IV	128	104 (53.89)	24 (35.82)			
**Angiolymphatic Invasion**				0.7806		
No	139	104 (61.54)	35 (63.64)		0.91 (0.62, 1.35)	0.6461
Yes	85	65 (38.46)	20 (36.36)			
**Perineural Invasion**				0.3618		
No	153	112 (66.67)	41 (73.21)		0.81 (0.50, 1.31)	0.3841
Yes	71	56 (33.33)	15 (26.79)			
**Histological Type**				0.1926		
Well and Moderately Differentiated	21	18 (10.65)	3 (5.00)		0.45 (0.14, 1.44)	0.1793
Poorly Differentiated	208	151 (89.35)	57 (95.00)			

Values expressed as frequency (%). * Adjusted for sex, tumor location, BMI, and smoking status. ^†^ Adjusted prevalence ratio for T Stage (T3 + T4 vs. T1 + T2). ^‡^ Odds ratio from the cumulative logistic regression model for Tumor Stage (I and II; III; IV). Adjusted for sex, tumor location, BMI, and smoking status. *p*-value calculated using the Chi-square test.

**Table 5:** Crude and adjusted hazard ratios according to Cox regression models for overall survival.

Variables	Crude Hazard Ratio (95% CI)	*p*-Value	Adjusted Hazard Ratio (95% CI) *	*p*-Value
**Sex**		0.2352		
Female	1			
Male	1.53 (0.76, 3.11)	0.2352		
**Tumor Location**		0.4862		
Rectum	1			
Colon	1.32 (0.60, 2.89)	0.4862		
**ASA Classification**		0.3840		
I and II	1			
III and IV	1.45 (0.63, 3.32)	0.3840		
**BMI**		0.0785		0.0477
<30	1		1	
≥30	2.23 (0.91, 5.48)	0.0785	2.51 (1.01, 6.24)	0.0477
**CEA**		0.4241		
≤5	1			
>5	1.35 (0.65, 2.79)	0.4241		
**Surgical Approach**		0.0688		0.0391
Laparoscopic	1		1	
Open	1.93 (0.95, 3.90)	0.0688	2.14 (1.04, 4.40)	0.0391
**Tumor Stage**		0.3301		
I and II	1			
III	1.72 (0.82, 3.63)	0.1511		
IV	1.05 (0.30, 3.72)	0.9339		
**Age**		0.6162		0.5375
<70	1.26 (0.51, 3.06)	0.6162	1.33 (0.54, 3.26)	0.5375
≥70	1		1	

* Adjusted for surgical approach, BMI, and age.

**Table 6:** Crude and adjusted hazard ratios according to Cox regression models for disease-free survival.

Variables	Crude Hazard Ratio (95% CI)	*p*-Value	Adjusted Hazard Ratio (95% CI) *	*p*-Value
**Sex**		0.8109		
Female	1.07 (0.63, 1.81)	0.8109		
Male	1			
**Tumor Location**		0.6885		
Rectum	1			
Colon	1.12 (0.63, 1.99)	0.6885		
**BMI**		0.0418		0.0464
<30	1		1	
≥30	2.19 (1.03, 4.68)	0.0418	2.09 (1.01, 4.46)	0.0464
**Surgical Approach**		0.3827		
Laparoscopic	1			
Open	1.27 (0.74, 2.16)	0.3827		
**Tumor Stage**		0.1468		
I and II	1			
III	1.47 (0.87, 2.49)	0.1468		
**CEA**		0.0592		0.0473
≤5	1		1	
>5	1.70 (0.98, 2.94)	0.0592	1.82 (1.00, 3.00)	0.0473
**T Stage**		0.8395		
T1 + T2	1			
T3 + T4	1.06 (0.60, 1.88)	0.8395		
**N Stage**		0.0989		
N0	1			
N1 + N2	1.56 (0.92, 2.63)	0.0989		
**ASA Classification**		0.2566		
I and II	1			
III and IV	1.42 (0.77, 2.60)	0.2566		
**Age**		0.1638		0.1939
<70	1.66 (0.81, 3.40)	0.1638	1.61 (0.79, 3.29)	0.1939
≥70	1		1	

* Adjusted for BMI, CEA, and age.

## Data Availability

In compliance with Brazil’s General Personal Data Protection Law (LGPD, Law No. 13709/2018), the dataset used in this study is not publicly available. However, it may be requested from the corresponding author, subject to ethical approval.
